# Implant Choice of Internal Fixation for Stable Femoral Neck Fractures in Elderly: Cannulated Screw Fixation versus Helical Blade Cephalomedullary Nailing

**DOI:** 10.5704/MOJ.2503.013

**Published:** 2025-03

**Authors:** YH Roh, JS Ahn, CM Lim, KW Nam

**Affiliations:** 1 Department of Orthopaedic Surgery, Jeju National University Hospital, Jeju, South Korea; 2 Department of Orthopaedic Surgery, Jeju National University, Jeju, South Korea; 3 Department of Orthopaedic Surgery, Uijeongbu Eulji Medical Center, Uijeongbu, South Korea

**Keywords:** femoral neck fracture, cephalomedullary nail, cannulated screw, internal fixation

## Abstract

**Introduction:**

The incidence of femoral neck fractures (FNFs) in elderly patients is increasing as average lifespans and the prevalence of osteoporosis increase. The optimal treatment strategy remains unclear. We compared the outcomes of cephalomedullary nail (CMN) and cannulated screw (CTS) fixations used to treat stable FNFs in patients over 65 years of age.

**Material and Methods:**

Among elderly patients with Garden type 1 and 2 FNFs treated between January 2010 and May 2018, 44 who were followed-up for more than 1 year were included. There were 28 cases in the CTS group and 16 cases in the CMN group, and the average age at the time of surgery was 76.3 years (range, 65–88 years). Radiological and functional variables were analysed to compare the results by fixation device.

**Results:**

There were no significant differences between the groups in terms of functional outcomes or bone union times. However, operation and fluoroscopy times were significantly shorter in the CMN group. The neck shaft varus angulation and the extent of device sliding were greater in the CTS group. Multivariate analysis showed that CTS use was independently associated with major complications.

**Conclusion:**

The CMN is a useful tool for treating stable FNFs in the elderly. It is simpler to use than conventional CTS fixation and is associated with fewer complications.

## Introduction

Femoral neck fractures (FNFs) are the most prevalent fractures in elderly patients, associated with high mortality and morbidity^1,2^. Many studies have evaluated the epidemiology of FNFs and the clinical results after treatment of such injuries^3-6^. The 1-month mortality associated with FNFs is about 10% and the 1-year mortality is about 30%. More than half of all elderly patients treated for FNFs are cognitively impaired^4,7^.

The treatment options for FNFs have been well-studied. Fracture site displacement, patient age, comorbid disorders, and pre-fracture activity level are important when surgeons consider how to treat them^8^. Stable FNFs (type I or II of the Garden classification)^9^ are usually further stabilised via internal fixation, and exercise commences early^10^. Internal fixation using multiple cannulated screws (CTSs) is frequently employed because it offers several advantages including fracture fixation stability, a low complication rate, and the ease of operation when using an image amplifier^11^. However, some authors have reported high rates of reoperation in older patients with poor bone quality; screw fixation alone may not provide adequate support^12-14^. For these reasons, some surgeons have considered a cephalomedullary nail (CMN) fixation strategy for problematic FNFs^15^. Theoretically, such devices ensure biologically friendly fixed-angle support for patients with difficult injuries.

In this study, therefore, we compared the results of CMN fixation and multiple CTS fixations of stable FNFs in patients over 65 years of age.

## Materials and Methods

**Fig. 1:**
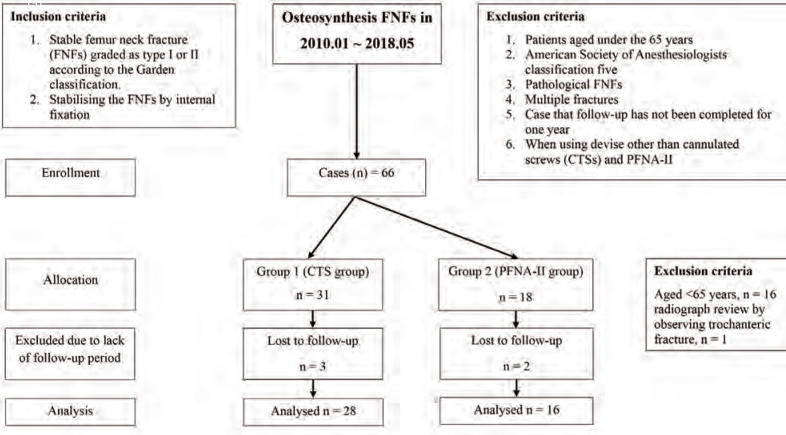
Patient enrolment flow diagram. This study involved 44 patients.

The Institutional Review Board of Jeju National University Hospital approved our request to search the surgical database of that institution to identify cases for the current study. Patients considered ineligible included those under the age of 65 years; those of American Society of Anaesthesiologists (ASA) classification 516; and patients with pathological FNFs, with multiple fractures, or those not followed-up for 1 year. A total of 66 patients underwent internal fixation of stable FNFs (Garden type I or II) from January 2010 to May 2018; 50 were aged 65 years or older and were considered for this study. Of the 50 patients, 2 were excluded because of death within 1 year from underlying disease, not as a consequence of fracture surgery. One patient was excluded because a plain radiograph revealed an inter-trochanteric fracture, and three patients were lost to outpatient follow-up (Fig. 1).

The remaining 42 patients (10 men and 32 women) were of mean age 76.1 years (range, 65–88 years). The mean body mass index (BMI) was 22.3kg/m^2^ (range, 15.6–33.2kg/m2). The bone mineral density (BMD) of the healthy hip was measured using dual energy radiograph absorptiometry, and the T-scores served as references. The average lowest T-score was –3.0 (range, –0.7 to –4.5); the score was below –2.5 in 31 cases and above –2.5 in 13 cases. Most fractures were attributable to low-energy injuries (slips or falls in 42 cases and minor motor vehicle accidents in 2). There were 34.1% (n=15) subcapital fractures of AO classification 31-B1, and 65.9% (n=29) transcervical fractures of AO classification 31-B2. According to the Garden classification, 31 patients had type I fractures and 13 had type II fractures. Walking ability was assessed using the Koval method^17^ from the preoperative period to 12 months after surgery and was graded from independent community ambulatory (grade 1) to nonfunctional ambulatory (grade 7) status. The average score increased from 1.77 pre-injury to 2.39 at the 12-month final follow-up. The mean ASA score was 2.0 (range, 1–3) and the mean follow-up period was 27.1 months (range, 13– 67 months).

In all cases, closed reductions were performed under traction on a fracture table, and reduction was confirmed using an image intensifier. Surgery was performed within 24 h of injury except when patients exhibited a high anaesthesia risk attributable to underlying medical conditions. Prophylactic antibiotics were administered to all patients pre-operatively. Fixations of fractures in group 1 were conducted between 2010 and 2014 using three or four CTSs and fixations of fractures in group 2 were conducted starting in 2015 using helical blade CMNs. All operations were performed by a single orthopaedic surgeon (NGW).

**Fig. 2:**
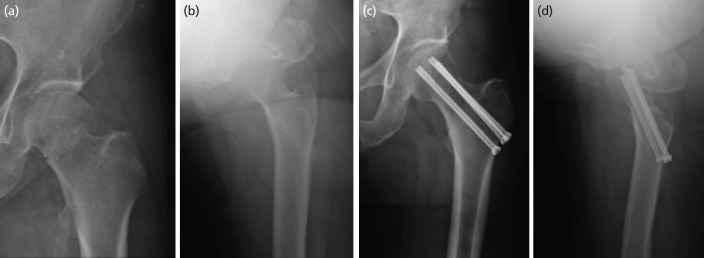
Plain radiological antero-posterior view and (a) translateral view, (b) of a valgus impacted femoral neck fracture of the left hip in a 67-year-old woman. Antero-posterior view, and (c) translateral view, (d) after cannulated screw fixation.

Each patient was placed supine and then the upper body was slightly supinated to the opposite side; this advanced the hip joint forward. An image amplifier was used when reducing fractures, and reduction status was based on the Garden alignment index^18^ of the opposite hip joint. For Garden type I fractures (valgus impacted fractures), in situ fixation was performed without attempting to reduce the fracture. The fractures in group 1 patients were fixed using 6.5mm partially threaded CTSs. Three 6.5mm or 7.0mm CTSs were fixed whenever possible to allow the screws to converge and diverge within 5° in the form of an inverted triangle. Then, depending on bone quality, a single CTS was placed, or a washer was used (Fig. 2).

**Fig. 3:**
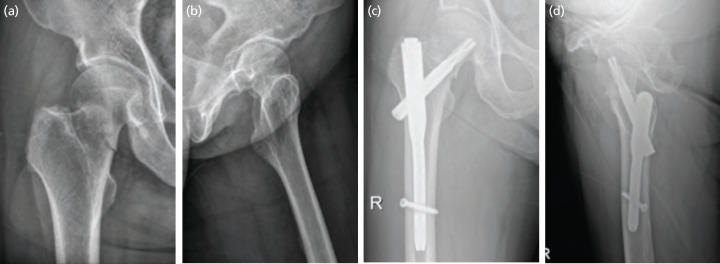
(a) Plain radiological antero-posterior view, and (b) translateral view of a valgus impacted femoral neck fracture of the right hip in a 73-year-old man. (a) Antero-posterior view, and (d) translateral view after cephalomedullary nailing.

In group 2 patients, a CMN, Proximal Femoral Nail Antirotation II device [PFNA-II; DePuySynthes®, Eimattstrasse 3, 4436 Oberdorf, Switzerland] was used for fixation. The size of the CMN was determined by reference to radiological images obtained before surgery. The angle of the cephalic blade was set by reference to the caput-column-diaphysis angle of the patient and the blade was secured in a position that was as central as possible but not facing upwards. The nail length was between 170mm and 200mm (Fig. 3).

Sitting was allowed from the first post-operative day, and wheelchair usage and partial weight-bearing commenced between post-operative days 3 to 7 depending on the extent of reduction, the patient’s systemic condition, and the extent of pain. Partial weight-bearing with a walker was allowed from the second post-operative week. Weight-bearing was gradually increased as fracture union proceeded, as revealed by radiography.

One of our objectives was to compare the clinical outcomes of CTS and CMN fixation used to treat FNFs. Clinical outcomes were determined by deriving Harris hip scores (HHSs)19. Patients completed questionnaires at 6 weeks, 3 months, 6 months, and 12 months after surgery. The surveys were administered by a trained research nurse blinded to clinical information who was not involved in the study. The operation time, image amplifier time, length of hospital stays, intra-operative bleeding and transfusion status, and Koval classification at 12 months after surgery were noted.

**Fig. 4:**
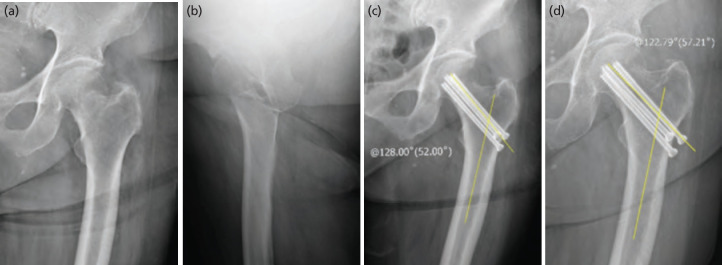
(a) Plain radiological antero-posterior view, and (b) translateral view of a valgus impacted femoral neck fracture of the left hip in a 75-year-old woman. (c) Neck shaft angle measured in antero-posterior view immediately after cannulated screw fixation, and (d) at 12 months.

**Fig. 5:**
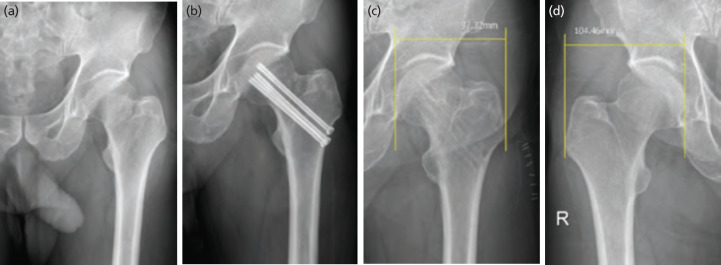
(a) Plain radiological antero-posterior view of a undisplaced femoral neck fracture of the left hip in a 66-year-old man. (b) Immediately after cannulated screw fixation. (c) Horizontal length of the left hip joint measured after obtaining bony union. (d) Horizontal length of right hip joint without surgery.

Implant-specific complications, implant positions, and fracture unions were evaluated using plain radiographs taken immediately after operation; at 6 weeks, 3 months, 6 months, and 12 months post-operatively; and then annually. Radiological fracture union was defined as bridging of the fracture site by callus or bone at a minimum of three cortices. Cortical healing was assessed in four anatomically proximal femoral regions (anterior, posterior, medial, and lateral) using anteroposterior and lateral plain hip joint radiographs^20,21^. Changes in neck shaft angle (NSA) were measured using the technique described by Paley^22^; a plain radiograph taken immediately after surgery was compared to a radiograph obtained 12 months after surgery (Fig. 4). Implant sliding distance was defined as the distance between the lateral cortical bone of the femur and the lateral tip of the blade or screw as observed via anteroposterior radiography^23^. To assess decreases in abductor moment arms, the horizontal length difference between the operated and unoperated sides was derived by measuring the distance from the medial border of the femoral head to the lateral border of the greater trochanter in the final follow-up radiograph taken after bone union^24^ (Fig. 5). Non-union was defined as a failure to achieve union by 12 months after surgery. Avascular necrosis (AVN) was classified radiologically using the method of Ficat^25^. The major complications were considered postoperative non-union, femoral head AVN, and reoperation. Radiological assessments were performed by two independent observers (CML and JSA), and mean values were calculated. Evaluations of and measurements from plain radiographic images employed a picture archiving and communication system [PACS; INFINITT, Infinitt Healthcare®, Seoul, Korea].

The clinical characteristics and surgical factors that varied continuously in the CTS group and the CMN group were compared using the student t-test and Mann-Whitney test. By contrast, the chi-square test and Fisher’s exact test were used to compare categorical variables. Furthermore, linear-by-linear associations with 95% confidence intervals were employed in univariate analyses that assessed the individual factors associated with major complications. Multiple logistic regression analyses were performed to identify independent predictors of major complications. All statistical analyses were conducted using IBM SPSS Statistics version 20.0 [IBM Co., Armonk, NY, USA]. Statistical significance was defined as a p-value <0.05.

## Results

In all, 42 elderly patients with 44 FNFs were treated; group 1 included 28 cases and group 2 included 16 cases. Demographic characteristics including sex, age, BMI, bone quality, BMD, the mechanism of injury, AO, Garden classifications, the Koval classification before injury, ASA risk score, and the follow-up period did not significantly differ between the two groups (Table I).

**Table I:** Baseline demographic characteristics of the patients in each group.

Characteristic	CTS group (N = 28)	PFNA-II groups (N = 16)	p-value
Sex (n, %)			
Male	5 (17.9)	3 (18.8)	0.689
Female	23 (82.1)	13 (81.2)	
Age (years)			0.691
Mean (range)	75.7 (65–87)	76.6 (66–88)	
Bone quality (n, %)			0.691
Normal	1 (3.6)	0 (0)	
Osteopenia	9 (32.1)	3 (18.8)	0.144
Osteoporosis	18 (64.3)	13 (81.2)	0.127
BMD (T score: g/cm^2^)	–2.8 ± 0.93†	–3.2 ± 0.82†	
Mechanism of injury			
MVA	0 (0)	2 (12.5)	
Fall	28 (100)	14 (87.5)	0.557
AO classification (n, %)			
31-B1	8 (28.6)	7 (43.8)	0.761
31-B2	20 (71.4)	9 (56.2)	
Garden classification (n, %)			0.749
Type 1	20 (71.4)	11 (68.8)	
Type 2	8 (28.6)	5 (31.2)	0.536
Koval before injury			
Mean (range)	1.61 (1–6)	2.06 (1–6)	
ASA score			
1 or 2	18 (64.3)	9 (56.2)	
3 or 4	10 (35.7)	7 (43.8)	
Follow-up (months)			
Mean (range)	28.9 (12.4–67.4)	25.4 (12.5–61.1)	

• CTS: Cannulated screw, PFNA-II: Proximal femoral nail anti-rotation II, BMI: Body mass index, BMD: Bone mineral density, MVA: Motor vehicle accident, Koval: Koval classification, ASA: American Society of Anaesthesiologists. • †The values are presented as means ± standard deviations.

There were no statistically significant differences in intra-operative bleeding or transfusion status, or hospital stay. In addition, there were no differences in the measured HHS. At 12 months of follow-up, the mean total HHSs were 75.2±14.3 and 81.9±15.2 in group 1 and group 2, respectively. The mean difference in the total HHS of the two groups was 6.7±3.7 (p=0.058). The HHS, pain score, functional and deformity status, and range of motion did not significantly differ between the two groups. In assessments of walking ability using the Koval classification, the average scores increased from 1.6±1.2 and 2.1±1.6 pre-injury to 2.4±2.1 and 2.4±1.60 at the 12-month final follow-ups in group 1 and group 2, respectively. However, statistically significant differences were observed in terms of the mean operation time (group 1, 57.6±11.1 min vs. group 2, 50.6±10.9 min; p=0.031) and the image amplifier time (group 1, 114.5±24.5 s vs. group 2, 83.9±24.1 s; p=0.000) (Table II).

**Table II:** Intra- and post-operative variables of the patients in each group.

Characteristic	CTS group (N = 28)	PFNA-II groups (N = 16)	p-value
Operation time (min)			
Mean (range)	57.6 (38–80)	50.6 (25–73)	0.031
Image amplifier time (s)			
Mean (range)	114.5 (79–153)	83.9 (58–119)	< 0.001
Intra-Op bleeding (mL)			
Mean (range)	157.0 (90–250)	168.1 (80–450)	0.693
Transfusion (mL)			
Mean (range)	428.6 (0–1,200)	487.5 (0–1,200)	0.679
Hospital stays (days)			
Mean (range)	20.1 (9–48)	25.3 (8–54)	0.091
HHS			
Total (0–100)	75.2 (62–93)	81.9 (65–97)	0.058
Koval at 12 months			
Mean (range)	2.4 (1–6)	2.4 (1–6)	0.896
Union period (months)			
Mean (range)	6.2 (3–9)	5.7 (3–9)	0.348
NSA change (°)			
Mean (range)	5.4 (0.0–11.5)	2.5 (1.4–6.0)	0.003
Device sliding (mm)			
Mean (range)	10.3 (2.0–21.4)	13.5 (3.7–23.0)	0.027
Horizontal shortening (mm)			
Mean (range)	5.4 (0.0–18.0)	4.2 (0.69–16.0)	0.352
Non-union (n, %)	2 (7.1)	0 (0.0)	0.526
Avascular necrosis (n, %)	4 (14.3)	1 (6.3)	0.393
Total complications (n, %)	8 (28.6)	1 (6.3)	0.063
Reoperation (n, %)	6 (21.4)	1 (6.3)	0.124

• CTS: Cannulated screw, PFNA-II: Proximal femoral nail anti-rotation II, Intra-Op: Intra-operative, HHS: Harris hip score, Koval: Koval classification, NSA: Neck shaft angle

Bone union at the fracture site was noted in 42 of the 44 cases (95.5%) at the final follow-up, and the average time to bone union was 6.1 months. There were no statistically significant between-group differences in bone union time (group 1, 6.2 months vs. group 2, 5.7 months; p=0.348) or the extent of horizontal shortening. However, statistically significant differences were observed in terms of NSA changes and implant sliding distance. In all cases, NSA changes affected varus angulation, which was significantly larger in group 1 than in group 2 (group 1, 5.4º vs. group 2, 2.5º ; p=0.003). The mean implant sliding distance was 10.3 mm in group 1 and 13.5 mm in group 2 (p=0.027).

**Fig. 6:**
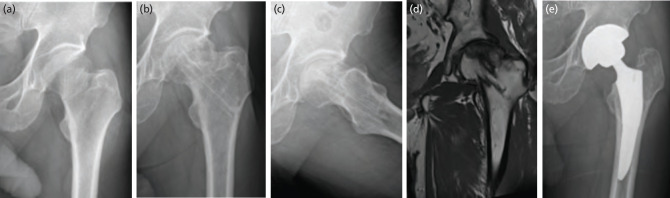
(a) Plain radiological antero-posterior view of a undisplaced femoral neck fracture of the left hip in a 66-year-old man. (b) Four-years after surgery, loss of sphericity of the femoral head was observed on anteroposterior view, and (c) translateral view. (d) Avascular necrosis of the femoral head was observed on hip magnetic resonance imaging proton density coronal view. (e) Conversion surgery to total hip replacement was performed.

**Fig. 7:**
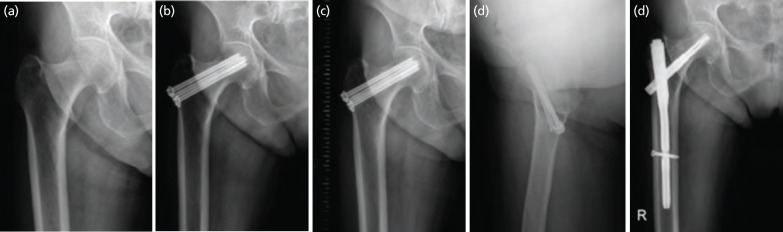
(a) Incomplete fracture in the supero-lateral area of the right femoral neck in a 76-year-old woman was observed, and (b) cannulated screw fixation performed. (c) Six-months subtrochanteric fracture observed below the cannulated screw without trauma in antero-posterior view and (d) translateral view. (e) Conversion surgery to cephalomedullary nailing was performed.

In group 1, two patients (7.1%) experienced non-union and thus conversion to arthroplasty. A further four patients (14.3%) exhibited changes in AVN, two of which were mild (Ficat stage II) and were thus treated conservatively. The other two patients developed severe AVN (Ficat stage III) and underwent arthroplasty (Fig. 6). Another complication was subtrochanteric fracture in two patients (7.1%). These patients were treated by changing the CTSs to a CMN that covered the fracture site (Fig. 7). In group 1, a total of six patients (21.4%) required reoperation during the follow-up period. On the other hand, in group 2, none of the 16 patients exhibited non-union (0.0%) and only one patient (6.3%) developed AVN that required arthroplasty (Ficat stage III). Although there were no statistically significant differences in the reoperation rate or the incidence of major complications between the two groups, the frequencies of both tended to be higher in group 1 than in group 2 (Table II).

Univariate analysis was employed to assess the individual effects of variables on major complications in all patients. No statistically significant differences were observed between the two groups in terms of any demographic, surgical, or radiological variable or the implant type, but the latter exhibited a trend (p=0.061) (Table III). Multiple logistic regression analysis seeking independent factors associated with several variables and major complications showed that the implant type was an independent predictor of major complications (p=0.048) (Table IV).

**Table III:** Univariate comparative analysis in terms of major complications.

Variable	No complication (N = 35)	Complication (N = 9)	p-value	Odds ratio	95% CI for the odds ratio
Sex (n, %)					
Male	6 (18.8)	2 (16.7)			
Female	29 (81.2)	7 (83.3)			
Age (years)	74.7 ± 4.1^†^	75.0 ± 0.9^†^	1000		
BMI (kg/m2)	22.0 ± 4.0^†^	23.3 ± 5.0^†^	0.797		
BMD (T-score)	-3.0 ± 0.9^†^	-2.8 ± 0.8^†^	0.361		
AO classification			0.652	1.154	0.199–6.698
31-B1	13 (37.1)	2 (22.2)	0.181	2.263	0.871–9.052
31-B2	22 (62.9)	7 (77.8)			
Garden classification			1.000	0.733	0.163–3.304
Type 1	25 (71.4)	6 (66.7)			
Type 2	10 (28.6)	3 (33.3)			
ASA score			0.343	1.909	0.497–7.337
1 or 2	23 (65.7)	4 (44.4)	0.061	0.163	0.021–1.015
3 or 4	12 (34.3)	5 (55.6)			
Device type			0.535		
CTS	20 (57.1)	8 (88.9)	0.565		
PFNA-II	15 (42.9)	1 (11.1)	0.612		
NSA change (°)	4.2 ± 3.1^†^	4.8 ± 2.9^†^			
Device sliding (mm)	11.2 ± 4.9^†^	12.2 ± 4.6^†^			
HS (mm)	5.1 ± 4.1^†^	4.3 ± 5.2^†^			

• CI: Confidence interval, BMI: Body mass index, BMD: Bone mineral density, ASA: American Society of Anaesthesiologists, CTS: Cannulated screw, PFNA-II: Proximal femoral nail anti-rotation II, NSA: Neck shaft angle, HS: Horizontal shortening. • ^†^Values are expressed as means ± standard deviations.

**Table IV:** Multivariate comparisons by incidence of major complications.

Variable	Exp (B)	95% CI of the odds ratio	p-value
Sex	0.994	0.088–11.274	0.996
Age	1.237	0.936–1.634	0.135
BMI	1.066	0.867–1.312	0.545
BMD	1.334	0.419–4.250	0.626
Garden classification	4.043	0.233–70.244	0.338
ASA Score	1.891	0.322–10.769	0.473
Device type	0.142	0.017–0.918	0.048

• CI: Confidence interval, BMI: Body mass index, BMD: Bone mineral density, ASA: American Society of Anaesthesiologists.

## Discussion

FNFs are frequent in elderly patients; about 50% are of Garden types I and II, thus undisplaced FNFs^26^. However, the optimal treatment for elderly patients with undisplaced FNFs remains very controversial given that the patient’s medical condition may be poor and also the risk imparted by anaesthesia. Handoll *et al*^27^ found that, in patients with undisplaced FNFs, surgical treatment was associated with more rapid recovery than conservative treatment and prevented conversion of undisplaced FNFs into displaced FNFs. In addition, the guidelines of the German Society for Traumatology recommend osteosynthesis of undisplaced FNFs regardless of patient age or bone quality^28,29^. In this study, we compared the well-known “inverted triangle CTS” osteosynthesis method for undisplaced FNFs, and fixation using PFNA-II, a CMN-based intramedullary device.

CTS fixation is relatively easy and rapid and has been widely chosen as the first option when surgically treating patients with undisplaced FNFs^13^. However, in our study, the average surgical time was 57.6 min in group 1 and 50.6 min in group 2. Thus, the surgery time was significantly shorter in group 2 (p=0.031), and the image amplification time was also significantly shorter at 83.9 s in group 2 compared to 114.5 s in group 1 (p<0.001). Thus, the radiation exposure of patients and medical staff was lower in group 2. These results show that CMN fixation is easier and more convenient than CTS fixation; the latter requires direct manual manipulation using a guiding mechanism. In addition, fixing of CTSs in the form of an inverted triangle is technically difficult because each guide pin must be fixed in parallel. If inexperienced surgeons frequently adjust a guide pin, this may weaken the entry point of the lateral femoral cortex, creating a risk of iatrogenic subtrochanteric fractures^30,31^. Patients with severe osteoporosis are at significant risk of femoral head damage if guide pins are frequently repositioned^32^. This may destroy the structural integrity of the subchondral bone, compromising the bone-holding power of the screw^33^. Therefore, in elderly patients with osteoporosis, it is best to select an implant that can be fixed with minimal manipulation.

CTS fixation prevents complications by not impairing the superior torsional stability or the femoral blood supply^34^. However, in patients with Pauwel type III FNFs with vertical fracture lines, it is difficult to ensure adequate stability of the fracture fragment using CTSs alone, given the strong shear forces^35^. East Asians have smaller and shorter femora that exhibit more lateral bowing than do the femora of Caucasians. In addition, the frequency of coxa-vara is very high if the femoral NSA is small^36^. In patients with small femora, it is difficult to fix three CTSs; the screws crowd together, imparting stress to the side wall. In addition, in cases with severe coxa-vara or lateral bowing, the trajectory of each CTS runs in the transverse direction, rendering it difficult to resist the vertical shear force. On the other hand, as an intramedullary device is located on the mechanical axis of the femur, CMN better resists vertical shear forces and the stress on the lateral wall is also less than that after fixation with CTSs^37,38^. In our study, the change in the NSA was smaller in group 2 than group 1, supporting the suggestion that CMN fixation is more resistant to vertical shear force than is CTS fixation. In addition, the PFNA-II was specially designed to include a helical blade, thus preserving the cancellous bone of the femoral head and preventing rotation of the fracture fragment, unlike conventional lag screws^39,40^.

A CMN does not always have advantages over CTS. The former device, like other intramedullary devices, requires splitting of the hip abductor prior to entry. The HHS and Koval classifications were determined to explore whether damage to the hip abduction mechanism affected the functional results after surgery. However, there were no differences in the clinical results according to implant type at 12 months after surgery. Min *et al*^41^ reported that a decrease in the abductor moment arm due to horizontal shortening of the femoral neck was directly related to both poor gait and reduced physical function. In this study, there were no differences in horizontal shortening between the two groups, and there were no differences in the clinical outcomes. Additionally, considering cost and availability aspects, CTS fixation is still an effective treatment method for non-displaced FNF. Therefore, various conditions must be taken into consideration when selecting a device.

In our study, CTS fixation in elderly patients with undisplaced FNFs was associated with a higher incidence of major post-operative complications compared to the use of CMN. The difference in surgical failure between the two groups was attributable to variation in the incidences of implant sliding. When a CTS is used, sliding of the proximal fracture segment is impossible; non-union may occur if reduction is not complete. In addition, if movement occurs at the fracture site, the NSA may be displaced toward the varus angle, increasing the risk of AVN. In our study, group I exhibited a smaller NSA and less implant sliding compared to group 2.

A strength of our research is that it was performed at a single centre. All operations were performed by the same skilled orthopaedic surgeon. Thus, the surgical outcomes were more consistent than those of works involving multiple orthopaedic surgeons at several centres. This reduced the number of variables that must be considered during treatment, increasing the reliability of the results. Another strength is that we used inferential statistical analysis to enhance the validity of the conclusions.

However, our work had certain limitations that need to be acknowledged and addressed. The main limitation was the retrospective design, creating a risk of observer bias. The quality and duration of follow-up were not standardised; some data were missing; and we could not control for confounding variables. In addition, the follow-up period was relatively short; we did not study the long-term results after treatment of undisplaced FNFs. Finally, the study population was relatively small.

## Conclusion

There were no differences in clinical outcomes when conventional CTS and CMN served as the fixation methods for osteosynthesis in elderly patients with nondisplaced FNFs. However, the surgical time and radiation risk were lower in the CMN group, and radiologically, changes in NSA were greater in the CTS group. The most prominent independent risk factor for major post-operative complications such as non-union, reoperation, and AVN was the CTS use.
